# Research and Development of Ankle–Foot Orthoses: A Review

**DOI:** 10.3390/s22176596

**Published:** 2022-09-01

**Authors:** Congcong Zhou, Zhao Yang, Kaitai Li, Xuesong Ye

**Affiliations:** 1 Sir Run Run Shaw Hospital, School of Medicine, Zhejiang University, 3 East Qingchun Road, Hangzhou 310016, China; 2 Key Laboratory for Biomedical Engineering of Education Ministry, Department of Biomedical Engineering, Zhejiang University, Hangzhou 310027, China

**Keywords:** ankle–foot orthoses, energy consumption, functional electrical stimulation, human in the loop

## Abstract

The ankle joint is one of the important joints of the human body to maintain the ability to walk. Diseases such as stroke and ankle osteoarthritis could weaken the body’s ability to control joints, causing people’s gait to be out of balance. Ankle–foot orthoses can assist users with neuro/muscular or ankle injuries to restore their natural gait. Currently, passive ankle–foot orthoses are mostly designed to fix the ankle joint and provide support for walking. With the development of materials, sensing, and control science, semi-active orthoses that release mechanical energy to assist walking when needed and can store the energy generated by body movement in elastic units, as well as active ankle–foot orthoses that use external energy to transmit enhanced torque to the ankle, have received increasing attention. This article reviews the development process of ankle–foot orthoses and proposes that the integration of new ankle–foot orthoses with rehabilitation technologies such as monitoring or myoelectric stimulation will play an important role in reducing the walking energy consumption of patients in the study of human-in-the-loop models and promoting neuro/muscular rehabilitation.

## 1. Introduction

Ankle joint injury is mainly caused by external forces or nervous system diseases such as hemiplegia. Particularly, stroke has the highest morbidity and fatality rate, there are 16 million people worldwide who suffer from strokes yearly and 6 million patients die from the disease [1]. Stroke patients with foot drop often exhibit a pattern of motion compensation that causes the slowing down of swing rhythm. At the same time, due to the shortened standing phase on the affected side, the energy consumption (EC) of walking is increased [2]. Ankle osteoarthritis (AO) affects more than 1% of the global population, and 70–80% of AO cases are caused by traumatic injury [3], which leads to long-term joint pain and decreased quality of life [4]. Severe ankle motor dysfunction could affect the patient’s lower limb motor ability, and increase the burden on family and society [5].

An ankle–foot orthosis (AFO) is applied to the ankle joint to improve walking ability, prevent or correct ankle–foot deformities, maintain the stability of lower limb joints, and enhance the load-bearing capacity of lower limbs [6]. It can also compensate for ankle–foot functions and promote the functional recovery of lower limbs through elastic materials or external forces [7]. In the case of muscle weakness, AFO provides auxiliary torques for dorsiflexion and plantarflexion. While in the case of muscle spasms, AFO provides limiting torques [8]. Appropriate orthotic design directly promotes the patient’s rehabilitation process, especially in restoring natural gait patterns [9]. AFO has attracted extensive attention from researchers since the 1970s. With more than 40 years of development, researchers have carried out a large number of targeted and innovative designs on the AFOs aiming at promoting lower limb rehabilitation. This review analyzes the design and development of AFOs from the perspective of improving walking ability and reducing walking EC, and it is concluded that the fusion of new AFO design and other rehabilitation technologies such as functional electrical stimulation (FES) may be expected to play a more important role in reducing EC in human in the loop and promoting neuromuscular rehabilitation.

## 2. The Design and Development of AFOs

### 2.1. Literature Review Strategy

The systematic review protocol was developed in accordance with the Preferred Reporting Items for Systematic Reviews and Meta-Analyses (PRISMA) statement.

#### 2.1.1. Search Strategy

Electronic database searches were performed from March 2022 to June 2022, conducted in Web of Science, IEEE Xplore, and PubMed Central according to search terms related to AFOs categories (Ankle Foot Orthosis*, Static ankle-foot orthoses*, fixed ankle-foot orthoses*, dynamic ankle-foot orthoses*, articulating ankle-foot orthoses*, non-articulating ankle-foot orthoses*, semi-active ankle-foot orthoses*) combined with lower extremity rehabilitation-related vocabulary (stroke*, foot drop*, foot inversion*, foot valgus*, gait cycle*, walking energy*, muscle activation*).

#### 2.1.2. Eligibility Criteria, Research Options, and Data Extraction

Studies of human participants of any sample size were eligible, and there were no age, gender, cultural, or ethnic restrictions. Studies must have investigated the use of any type of ankle–foot orthosis (static ankle–foot orthosis, fixed ankle–foot orthosis, dynamic ankle–foot orthosis, articulating ankle–foot orthosis, non-articulating ankle-foot orthosis, semiactive ankle–foot orthosis) on outcomes related to walking ability or biomechanical function, mechanical properties, patient comfort, pain, and disability. Any other type of orthoses (orthoses for ankle joints, hip and knee joints) or orthoses not used for walking (such as massage therapy) were excluded. Unpublished data and data from studies that were not fully published were excluded.

After duplicates were removed, two authors (C.Z.) and (Z.Y.) screened titles and abstracts from the search results using predetermined eligibility criteria. Full-text articles were searched and independently reviewed for inclusion by two authors (X.Y. and K.L.). Data extraction and evaluation of the remaining articles were then independently completed by two authors (C.Z. and Z.Y.). Data extraction included study design, design features, and experimental effects.

#### 2.1.3. Description of Included Studies

**Figure 1:**
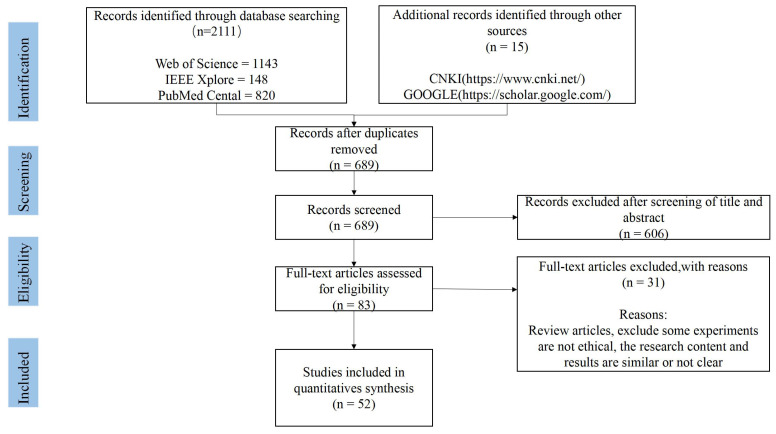
Flow diagram of the literature review process.

The initial electronic database search retrieved a total of 2126 articles, leaving 689 articles after deduplication. After completing the title and abstract screening, 83 articles were selected for possible inclusion in this review. After full-text screening, 52 studies met the inclusion criteria and were included in this review [10,11,12,13,14,15,16,17,18,19,20,21,22,23,24,25,26,27,28,29,30,31,32,33,34,35,36,37,38,39,40,41,42,43,44,45,46,47,48,49,50,51,52,53,54,55,56,57,58,59,60,61]. A flowchart of the search history and selection process is shown in Figure 1.

AFOs are usually designed from the shank to the sole of the foot and can maintain proper movement of the ankle joint. AFOs act on the shank and foot through the action of force to prevent foot drop, eversion, and inversion. The benefits of using AFOs are to help patients relieve physical pain and improve their self-care ability and quality of life. Scholars have also paid attention to utilizing AFOs to improve walking ability and reduce walking EC. Currently, new AFOs design mainly focus on the manufacture and combination with elastic materials or external dynamics.

### 2.2. Classification and Development

There are many types of orthoses at present. In 1992, the International Standardization Organization (ISO) defined AFOs with the nomenclature of orthosis assembly parts into ankle–foot orthoses (AFOs), knee-ankle–foot orthoses (KAFOs), and hip-knee-ankle–foot orthoses (HKAFOs) [62]. According to the different functional structures, AFOs can be divided into static AFOs, dynamic AFOs, and custom AFOs [63]. Recently, AFOs are divided into passive ankle–foot orthoses (PAFOs), semi-active ankle–foot orthoses (SAFOs), and active ankle–foot orthoses (AAFOs) according to whether the devices can directly provide power for walking [10,11].

**Figure 2:**
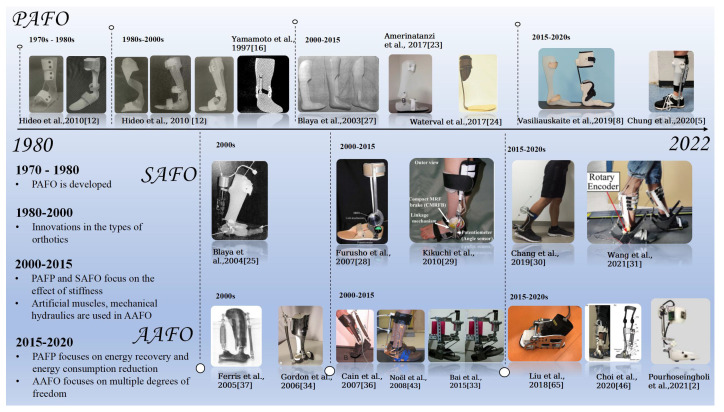
Classification and development trends of AFOs.

**Figure 3:**
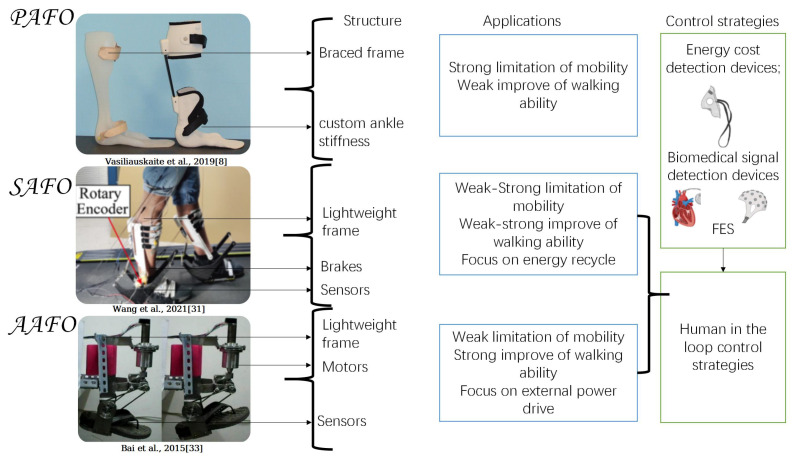
Structure, utilizations, and control strategies of AFOs.

This review will describe the detailed research and development process based on how the AFOs provide power. As shown in Figure 2, this includes: (1) PAFOs, which include static ankle–foot orthosis, partial hinged ankle–foot orthosis, and dynamic ankle–foot orthosis. The PAFOs proposed in this review are not comprised of electrical/electronic elements or power sources. They are usually comprised of mechanical elements such as dampers or springs; (2) SAFOs, which use brakes as control elements, such as active clutches and adaptive dampers. SAFOs can adaptively adjust joint impedance or recycle walking energy, but do not provide additional power for walking directly; (3) AAFOs, which are usually composed of a power supply, control system, sensors, and actuators. AAFOs can provide extra power directly for walking. Generally, PAFOs usually have a relatively simple structure and production process. They are mainly applied to limit the movement of the ankle joint, while PAFOs can store part of the energy generated by body movement in linear or spring elements, then release energy when needed to assist walking. The structure, utilizations, and control strategies of AFOs are shown in Figure 3. SAFOs and AAFOs can provide assistance for patients to walk by controlling actuators, and improve the ankle joint movement of patients with dysfunction caused by various injuries and neurological diseases. In recent years, researchers focus on how to improve walking ability and reduce walking EC by proper system design.

### 2.3. General Research and Development Processes of AFOs

**Figure 4:**
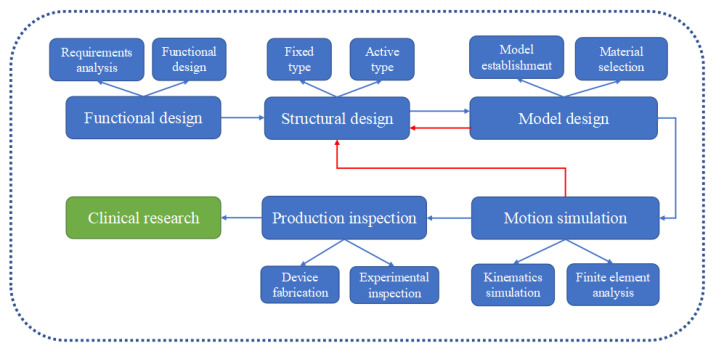
Design and manufactural processes of AFOs.

The design and manufactural processes of different AFOs categories are mainly consistent. In this section, this review summarizes and analyzes the general design and production processes of AFOs. As shown in Figure 4, the processes flow includes functional design, structural design, model design, motion simulation, production inspection [64,65], and clinical research [66]. Within these processes, structural design, model design, and motion simulation play significant and important roles in achieving reliable function and reaching the standards of clinical research. The detail design contents are concluded as follows.

(1) Functional design: Functional design process includes requirements analysis. The requirements analysis mainly focuses on understanding, analyzing, and sorting out the basic demands of the user. It can be divided into physical needs and psychological needs. The functional design of AFOs arecarried out on the basis of requirements analysis.

(2) Structural design: Structural design mainly refers to the determination of the overall structure of the AFOs. The overall structure may be divided into fixed type and active type. The fixed type generally plays the role of support, protection, fixation, and load relief. The active type could increase the range of motion of the ankle joint and assist the movement.

(3) Model design: The model design includes model establishment and material selection. The model establishment is mainly to obtain human body data through direct measurement or three-dimensional scanning, and then generate ankle models on the computer. Material selection is based on the function of each structure. The main materials are carbon fiber and synthetic plastics, occasionally alloys, foams, ceramics, and so forth.

(4) Motion simulation: It is important for the orthosis to work according to the functional design. Finite element analysis of the assembly, which provides the analysis of static structural strength and stiffness, should be performed. If the analysis results meet the strength and stiffness requirements, the product could be processed and produced. Otherwise, if the analysis results are not satisfactory, the structural design of the AFOs need to be re-carried out.

(5) Production inspection: Production inspection includes device fabrication and experimental inspection. The traditional fabrication method of AFOs adopts the method of injection molding, which uses a plate with a constant thickness that normally has a long production cycle. This method is difficult to iteratively optimize in the future [67,68]. On the other hand, 3D printing technology is based on intelligent digital models, it uses metal, plastic, and other adhesive materials to construct objects with layer-by-layer printing. It can be directly formed or customized and has great potential in the production of AFOs. After the production process, the orthosis is tested through material experiments which focus on evaluating the mechanical properties of the orthosis. The structural design needs to be re-carried out if it does not meet the standards.

(6) Clinical research: Clinical research usually recruits healthy volunteers or patients as experimental subjects to analyze the impact of AFOs on human walking ability, biomechanics, and walking EC through 3D motion capture equipment, EMG sensors, EC testers, and other instruments [69,70]. In addition, some studies have shown that AFOs combined with rehabilitation methods such as botulinum toxin and FES may have better effects on rehabilitation [71,72]. Some authors utilized botulinum toxin type A injection combined with an ankle–foot orthosis to improve the rehabilitation process of patients with post-stroke lower limb spasticity.

#### 2.3.1. Passive Ankle–Foot Orthoses (PAFOs)

As analyzed previously, PAFOs do not have any electronic control elements to control ankle motion during gait other than mechanical elements such as springs or shock absorbers. PAFOs can be subdivided into articulated devices and nonarticulated devices [10]. Passive non-articulating ankle–foot orthoses (PNAAFOs) are usually one piece that holds the ankle completely in one position. Passive articulating ankle–foot orthoses (PAAFOs) are designed to combine a lightweight thermoplastic or carbon composite shell with an articulating joint that allows a range of motion in the ankle joint. Articulated joints come in different designs with various hinges, flexion stops, and stiffness control elements such as springs, oil dampers, one-way friction clutches, and so forth.

Primevally, a large number of PNAAFOs were studied [12]. They were mainly designed to hold the ankle in one position and limit the mobility of plantarflexion thoroughly. However, the materials of the orthoses were stiff, which might result in excessive knee flexion moments during load response which resulted in unsteady walking. With the advancement of material science, the design of PNAAFOs gradually evolved from rigid to elastic. The characteristics of these orthoses mainly depended on the material and geometry [11,13,14]. Rear leaf spring orthoses were semi-rigid plastic orthoses that assisted toe flexion and prevented falls during the pre-swing period. Carbon fiber orthoses are another typical semi-rigid orthoses that can significantly improve pathological gait by storing energy during deformation and increasing thrust during the pre-swing period. Researchers have shown that carbon fiber orthoses can reduce energy expenditure in impaired patients [15].

PAAFOs appeared in large numbers in the 1980s and 1990s. The Okawa Ankle-Foot Orthosis was developed by Okawa Hara in 1981 [12] and provided some lateral stability through its lateral joints. Since then, articulated orthoses of different joint styles have sprung up one after another. At the beginning of the 21st century, articulated orthoses were continuously improved. In 1997, Yamamoto et al. [16] improved articulated orthosis with dorsiflexion assistance. A traditional AFO along with the Klenzak ankle joint was modified to prevent falls during walking. Their modified design added a spring to the rear of the orthotic tibia, which might create plantarflexion resistance when the heel touches the ground and prevent the foot from slipping. In 2002, Kawamura et al. [17] developed a passive mechanical element with variable elasticity and viscosity. The material was soft and light, and the element itself was small in size. The mechanical impedance of the element could be changed by adjusting the vacuum pressure applied to it. These characteristics made passive pneumatic components more convenient than active components of the wearable robot, such as electromagnetic, magnetorheological, or electrorheological brakes. Before the advent of oil dampers, orthoses were more likely to use spring dampers. In 2005, Yamamoto et al. [18] developed a small, lightweight hydraulic oil damper to provide torque resistance to plantarflexion. The oil damper absorbed the shock of heel impact and provided damping during load response.

Researchers have also studied the interaction between AFOs and the human body. Geboers et al. [19] studied ankle fixation and its effect on dorsiflexor strength, and their results showed that the use of AFO after nerve injury may lead to reduced dorsiflexor strength in a short period of time. Studies have shown that AFOs should provide horizontal resistance to flexion of the digits to simulate eccentric contraction of the dorsiflexors, thereby allowing a limited amount of loading response to act on plantarflexion [20]. A study by Hesse et al. [21] found that reduced dorsiflexor activity may lead to disuse atrophy and long-term dependence on orthoses. These studies suggested that insufficient orthotic stiffness may result in insufficient biomechanical control of ankle motion and excessive knee extension during gait, which in turn might lead to a stiff walking gait cycle, lower muscle activity, and muscle atrophy. Therefore, ankle-fixed AFOs, including PNNAFOs, might delay recovery in patients with neurological impairment.

In view of this, researchers have developed innovative AFOs with the motive of designing AFOs with predetermined stiffness or variable stiffness that meet the individual needs of patients. In 2015, Mataee et al. [22] proposed two technical solutions for the design of variable stiffness orthosis based on the mechanical and structural stiffness control of shape memory alloys. These designs could improve gait abnormalities in patients with foot drop for different walking conditions (e.g., different walking speeds). The first design modulated the torsional stiffness by controlling the axial load with the superelastic rod, and the other modulated the bending stiffness of the element by adjusting the effective length of the superelastic hinge. Although Mataee’s study effectively solved the problem of variable stiffness, it was difficult to control the shape-memory alloy components during cooling. Amirhesam et al. [23] found that the hyperelastic NiTi spring had nonlinear characteristics in elongation and compression. They hypothesized that the hinge could make the stiffness of the ankle more similar to that of a healthy person, which could help patients walk more naturally. On this basis, they focused on the performance of the hyperelastic NiTi spring and the transmission stainless steel spring and found that the NiTi spring could provide a wider range of motion and increased torque level. In addition to exploring the effect on gait, some studies also showed that the reduction in walking energy was related to the stiffness of the orthosis. Niels et al. [24] produced an AFO with adjustable stiffness using carbon fiber plate springs. For each patient, they assessed the walking energy costs, gait biomechanics, and walking speed of five AFOs with different stiffness. The results were used to determine the optimal stiffness for each patient.

In conclusion, PNAAFOs and PAAFOs are mainly distinguished by the degree of wrapping of the ankle and the structural design, as shown in Table 1. There are various types of PNAAFOs, and their main functions are to limit the movement of the ankle joint and provide support for the patient to walk. Although PNAAFOs can improve pathological gait to a certain extent and reduce walking EC, they limit the normal motion of the ankle joint. On the basis of the PNAAFOs, the PAAFOs provide a certain range of motion for the ankle joint through structural design. Both of PNAAFOs and PAAFOs can improve foot biomechanics and walking ability, reduce walking EC by adjusting joint stiffness, and enable patients to have a near-normal gait.

#### 2.3.2. Semi-Active Ankle–Foot Orthoses (SAFOs)

The motor control of PAFOs is limited by passive components as discussed while SAFOs and AAFOs have the ability to interact with the walking environment. SAFOs consist of electronic control systems, actuators, tethered or untethered powertrains, and stiffness control elements such as magnetorheological (MR) fluid brakes. Normal control systems typically include components such as force sensors, accelerometers, and microprocessors. Blaaya et al. [25,26,27] developed a SAFO with variable impedance based on elastic brakes. The elastic brake consists of a direct current motor, a mechanical connecting rod, and a torsion spring which could actively adjust the joint impedance of the ankle. The developed actuator weighed 2.6 Kg and required a bulky battery as a power supply. Furusho et al. [28,29] proposed installing an MR fluid brake at the ankle joint. The device could control the brake force by changing the intensity of the applied magnetic field, and it could provide a maximum resisting torque of 11.8 N·m. In addition, the authors applied the connecting rod mechanism to amplify the torque which was up to 24 N·m. Kikuchi et al. [29] further developed a more compact MR fluid brake. Compared with the previous prototypes, the proposed orthosis had a lighter weight, a more sensitive control system, and could assist ankle plantarflexion. SAFOs were further applied to recover gait energy during walking, provided assistance, and reduced walking EC. Chang et al. [30] developed an energy recovery system composed of a torsion spring and two actively controlled clutches to control the accurate time point of energy recovery and energy release. Wang et al. [31] developed a novel, lightweight heel strike energy storage mechanism including a clutch. They applied a series of springs that helped users reduce walking EC.

Table 2 demonstrates the comparison of SAFOs in mass and effects. The power assist control units within SAFOs are evolving in the direction of lightness and precise control. The weights of SAFOs are gradually reduced from 2.6 Kg to nearly 1 Kg, or even less than 800 g. This is a clear advantage of SAFOs over AAFOs. In terms of the assisting effects provided by SAFOs, the range of resistance torque that the device could provide should be studied first. Then, the benefits of SAFOs on human walking might be studied in the form of muscle activation during walking by myoelectric sensors and EC testing instruments directly.

#### 2.3.3. Active Ankle–Foot Orthoses (AAFOs)

Torque can be transmitted to the ankle by AAFOs using external energy and power units, while the orthosis may be adjusted by computer control to give the users a more natural gait [32]. Pneumatic muscles are characterized by light weight and high power, and are gradually being applied in the development of AAFOs [33]. As a typical representative, Ferris et al. [34,35,36,37] proposed an AAFO that could provide the torque required for toe flexion and dorsiflexion through two artificial pneumatic muscles. The device was relatively lightweight (1.6 Kg), and the user’s peak plantarflexion torque was reduced by 64% and the peak dorsiflexion torque was increased by 23% after wearing it. The experiment required an onboard power supply and computer assistance, which was suitable for laboratory research and rehabilitation. In view of the above-mentioned limits, Alex et al. [38] proposed a kind of pneumatic driven orthosis that might be used daily in the household. The device had a bidirectional rotating air motor at the ankle and a CO${}_{2}$ bottle with a regulator at the waist. The power supply was separated from the actuator to minimize the weight of the ankle. The experimental results showed that the system had an obvious auxiliary effect on functional plantarflexion. However, since the system could only provide 9 N·m of torque at rated power, it was mainly suitable for auxiliary plantarflexion.

With the deepening of research, hydraulic technology has also emerged in this research area. Compared with electromechanical systems, hydraulic technology has the advantages of high power and is only limited by the pressure of the working fluid [39,40]. Studies have shown that compared with the equivalent electromechanical system above 500 pounds per square inch (psi), the overall weight of the 100-watt hydraulic system is lighter [41]. Compared to electric motors, hydraulic systems have higher responsiveness and greater stiffness, enabling faster start-up and stops along with small position errors [39,42]. Brett et al. [32] designed a hydraulic AAFO which consisted of a hydraulic power source at the waist and a hydraulic brake at the ankle, connected by a pair of hoses. The weight of the ankle actuator and the power supply met the design requirements of 1.0 Kg for the ankle and 3.5 Kg for the waist. Although the total weight of the system was similar to the weight of the electromechanical system, lightweight hydraulic actuators could significantly decrease the ankle weight compared with the electromechanical system. Martin et al. [43] combined the characteristics of the electric and hydraulic systems, and designed an electro-hydraulic AAFO that could provide forward rotation of the ankle joint. Kim et al. [44] proposed a completely unconstrained pneumatic AAFO powered by a custom compressor, which miniaturized the compressor by optimizing the air compression rate to help foot-fall patients.

Studies have shown that the range of motion (ROM) of the ankle valgus is highly correlated with walking stability [45]. The ankle valgus maintains the center of pressure (COP) of the supporting foot and prevents the body from tilting to one side. Specifically, when the body is tilted, the misalignment between the projection of the center of gravity and the COP causes the tilting moment, and the subtalar joint could be rotated around the front surface to maintain the balance of the body. This move, known as the foot tilting strategy (FTS), produces stabilizing moments and returns the unstable body to a balanced position. Most studies of AAFOs have focused on sagittal motion, and they are useful in assisting with dorsiflexion, but not in valgus ROM. Choi et al. [46] designed a 2-DOF (degree of freedom) AFO by simulating the ankle joint and subtalar joint, and verified the performance of artificial pneumatic muscles used for balance training.

As demonstrated in Table 3, AAFOs and SAFOs have obvious differences in the way of providing walking assistance. SAFOs commonly use spring clutches, elastic actuators, and MR fluid as brakes. They provide assistance for walking by changing the stiffness of the ankle joint or recovering energy instead of providing assistance for plantarflexion and dorsiflexion directly. AAFOs usually use pneumatic artificial muscles, mechanical electric drives, and hydraulic methods to provide the torque of plantarflexion and dorsiflexion for human walking directly. The weight gradually decreases as a split design is usually applied to reduce the load-bearing of the ankle joint.

## 3. Discussion

The motion control units and potential effects of the discussed three types of AFOs are shown in Table 1, Table 2 and Table 3. PAFOs are widely applied in the field of ankle and foot rehabilitation because of their simple structural design and production process. However, PNAAFOs limit the movement of the ankle joint and are more effective in fixing the ankle and providing support for patients to walk, which have limitations when applied. Compared with non-articulating orthoses, articulated ankle–foot orthoses can adjust ankle stiffness by controlling springs, oil dampers, and magnetorheological fluid brakes, further improving biomechanics and promoting patient recovery. SAFOs and AAFOs can directly or indirectly assist patients in walking through electronic control systems, and they have advantages in improving walking ability and reducing walking energy consumption.

The development of orthoses, on the basis of the above-summarized structures and efforts, draws more attention to the integration with other rehabilitation technologies such as FES technology. Another development trend is as a part of walking assistance devices which are used for the study of walking ability and walking EC in human-in-the-loop models, and to explore new motion control strategies to further promote the motion recovery of single and multi-joint lower limbs.

### 3.1. Combined Study of AFOs and FES

For individuals with stroke or hemiplegia, walking ability is one of the most important indicators to evaluate the recovery of motor function. During the rehabilitation process, the joint movement pattern of the extensor muscles may cause abnormal gait such as foot drop, which affects walking efficiency and increases the risk of falling [47]. Studies have shown that the combination of AFOs and FES has a better effect on foot drop caused by upper motor neuron palsy, by installing electrodes locally on the AFOs and applying FES during walking. During this process, AFOs can control the joint mobility of the ankle joint to a certain extent, which helps to improve walking stability, while it may limit the plantarflexion of the ankle joint when the foot is off the ground and affect the walking speed [48,49]. FES can enhance the input stimulation of nerves and accelerate the establishment of cerebral collateral circulation without affecting the ankle plantarflexion when off the ground, which promotes the establishment of normal movement patterns [50,51]. The establishment of cerebral collateral circulation could reflect the rehabilitation status of patients with cerebral palsy. Early ankle dorsiflexion training and toe stimulation of peripheral sensory muscles can regulate the excitability of neurons in the neural reflex circuit, as well as promote the establishment of ankle dorsiflexion muscle responses. These rehabilitation strategies can improve the contractile load and muscle tension of related muscle groups and inhibit pathological gait such as foot drop [52].

Pagnussat et al. [53] assessed the effect of FES on the peroneal nerve on walking speed, ankle dorsiflexion range of motion, balance, and functional range of motion. Results showed that FES could improve ankle dorsiflexion, balance, and functional mobility. Nevisipour’s team [54] investigated: (1) the underlying biomechanical mechanisms of falls in chronic stroke patients using AFOs and FES for a long time; (2) the effects of AFOs and FES devices on the occurrence of falls in chronic stroke patients. The results showed that the AFOs/FES devices had a positive effect on static balance (balance ability during static motion) and could reduce the occurrence of falling events. It is necessary to explore methods and devices to enhance the establishment of dynamic balance (balance ability during dynamic motion) in the future. Khaghani’s team [55] compared the improvement of balance and walking ability in patients with multiple sclerosis (MS), a chronic progressive nervous disorder, by using FES alone and FES combined with AFOs. The results showed that under the condition of the AFOs equipped with the FES system, the patient’s postural response when walking back and forth was better than that of the FES system alone. In their study, only PNAAFO is used, while PAAFO, SAFO, and AAFO are expected to show better results in comparative studies in terms of rehabilitation.

Some other researchers focused on comparing the effects of AFOs and FES as separate rehabilitation methods on the establishment of static and dynamic balance, and comparing the advantages and disadvantages of the two methods in reducing walking EC and improving walking ability [56,57]. There was also research comparing the improvement of walking ability between FES alone and FES with PNAAFO, and the preliminary results verified that the fusion of the above two technologies could help improve the rehabilitation effect. However, there is still a lack of assessment and discussion on how FES and AFOs can be integrated, and the exploration of the sequence and method of FES application still needs to be further developed to reduce the occurrence of falls caused by long-term use [54].

### 3.2. Research on AFOs in Human in the Loop

In recent years, AFOs have played an important role in the study of human-in-the-loop control strategies. Prof. Collins’ team [58] designed an underactuated ankle exoskeleton. The device used a spring to simulate the Achilles tendon of the human body, which realized the energy storage and release at each stage of the human walking process and reduced the walking EC by 7.2%. Based on the idea of human in the loop, the assist torque was corrected through EC detection, and the target ankle joint assist curve was parameterized. By detecting the metabolic consumption of the human body, their team used the covariance matrix adaptive evolution strategy to adjust the parameters of the assist curve and iteratively generate the optimal assist curve, so that the metabolic consumption of the human body under the assistance of the exoskeleton was the lowest. The metabolic consumption was 24.2 ± 7.4% lower than that of the zero assist torque. Zhang’s team [59] presented 10 kinds of ankle walking-assist exoskeleton assist curves, and used the particle swarm algorithm to solve a set of optimal weight coefficient combinations of the activation degrees of different muscles as an evaluation function of the human in the loop.

The related research results showed that the use of the new evaluation function to optimize the power assist curve in the loop control of the human body could further reduce the degree of muscle activation during walking. Zachary’s team designed a real-time adaptive ankle exoskeleton controller capable of accurately assisting in a variety of walking conditions without the need for walking condition classification or real-time assessment of muscle activity, which provided the foundation for the application of AAFO in free-living situation [60]. However, the muscle coordination mode of the human body during walking can be changed to a certain extent affected by AFO, and then result in the compensatory phenomenon of some muscle groups. It is necessary for researchers to further study the theory of physical–physiological integration of human–computer interaction [61]. The problem of how to reasonably select the activation degree and weight of the lower limb muscles is still unsolved. A strategy that ensures the optimal labor saving achieved under the condition of AFOs assistance and maintains the original muscle coordination mode as much as possible should be studied in future work. To conclude, firstly, there are a series of studies focusing on how to map kinematics or kinetics parameters such as joint angles and torques from ‘superior’ bio-parameters such as located EEG signals and muscle synergies. These ‘superior’ bio-parameters can be obtained by a series of processing methods, such as blind source separation methods and over complete dictionary methods on collected EEG signals and sEMG signals to obtain sparsity features or features in other domains for data dimension reduction or a more accurate and robust mapping result. These features contain physiological factors so that, on one hand, they have a better real-time ability and a more compliant man-machine control strategy. On the other hand, they are closer to the physiological background of motion control strategies so they are normally appropriate for research on neural rehabilitation. Secondly, energy consumption during human activities such as walking has been fully researched in recent years. However, energy consumption relies on real-time dynamics gas component analysis techniques and devices which are commonly difficult to be used in real environments. More convenient energy consumption evaluation methods need to be further researched in the future.

## 4. Conclusions

In conclusion, this paper reviews the recent literature on the innovative design of AFOs, and discusses the development of PAFOs, SAFOs, and AAFOs. PAFOs have attracted attention since the 1980s and scholars have studied continuous designs for the shape and ankle joint styles of AFOs. With the advancement of clinical rehabilitation technology and the in-depth study of human walking gait, the further development of AFOs has been promoted from shape and style to material properties and muscle group responses. SAFOs and AAFOs have been studied since the early 21st century. Scholars focused on how to reduce the weight of the overall device and increase the portability and wearing experience of the device through different technical methods firstly, and then mainly focus on the role in the field of rehabilitation recently. In addition, it is also important to pay attention to the impact of joint movements other than the ankle so as to provide a new way for clinical rehabilitation training. However, the fundamental research on AFOs is still facing problems such as most experiments on AFOs focusing on the motion angle of the ankle joint, the moment of plantarflexion, and dorsiflexion while the muscle state and in-depth physiological indicators are rarely assessed accurately. Some studies have carried out experiments on the combination of AFOs, botulinum toxin, and FES while most of them are mechanical combinations, and the discussions on the mechanism are rare. In order to achieve smooth and labor-saving walking assistance, it is urgent to focus on breakthroughs in the AFOs elastic drive design and human-in-the-loop assist control technology to carry out theoretical research on the integration of human–computer interaction and physics–physiology integration theory. In addition to studying detailed materials and mechanical properties, innovative AFOs also need to be combined with other clinical rehabilitation methods to provide new ideas and methods for patient rehabilitation.

## Figures and Tables

**Table 1:** Comparison of features and functions between PNAAFOs and PAAFOs.

Category	Device Name/Author	Design Features	Effects	Ref.
PNAAFOs	Ortholen drop foot brace	Half wrap ankle	1. Fix ankle	[12]
			2. Provide lateral stability	
	Ortop AFO LH	No wrap ankle	1. Limit plantarflexion	[12]
			2. Provide lateral stability	
	Finer AFO	Full wrap ankle	1. Fix ankle	[12]
			2. Provide lateral stability	
PAAFOs	Okawa, H	Simple hinge	1. Promote dorsiflexion	[12]
			2. Limit plantarflexion	
			3. Provide lateral stability	
	Yamamoto, S	Spring	1. Reduce knee hyperextension	[16]
			2. Increase walking speed	
			3. Adjust the dorsiflexion auxiliary moment	
	Yamamoto, S	Oil Damper	1. Promote dorsiflexion	[18]
			2. Correct varus/valgus	
			3. Adjust orthosis stiffness	
	Mataee, M	Shape Memory Alloys	1. Improve biomechanics	[22]
			2. Promote normal plantarflexion	
	Amerinatanzi, A	Superelastic NiTi Spring	1. Greater range of motion	[23]
			2. Promote normal plantarflexion	
	Waterval, N	Customed spring	1. Reduce walking EC	[24]
			2. Improve biomechanics	
			3. Increase walking speed	

**Table 2:** Comparison of SAFOs in mass and effect.

Author	Motion Control Elements	Mass	Effect	Ref.
Blaya, J	Series Elastic Actuator	2.6 Kg	——	[25]
Furusho, J	Magnetorheological Fluid	1.6 Kg	Provide 24 N·m resistance torque	[28]
Kikuchi, T	Magnetorheological Fluid	0.99 Kg	Provide 10 N·m resistance torque	[29]
Chang, Y	Spring Clutch	0.9 Kg	10–20% decrease in gastrocnemius muscle activation	[30]
Wang, C	Spring Clutch	0.754 Kg	6% reduction in metabolic cost	[31]

**Table 3:** Comparison of AAFOs in mass and effect.

Author	Motion Control Elements	Mass	Effect	Ref.
Neubauer, B	Hydraulic boost	1 Kg at the ankle, 4.5 Kg at the Waist	Maximum 60 N·m auxiliary torque	[32]
Ferris, D	Artificial Pneumatic Muscle	Total weight 1.7 Kg	64% reduction in peak plantarflexion torque and 23% increase in peak dorsiflexion torque	[35]
Cain, S	Artificial Pneumatic Muscle	——	53% reduction in peak plantarflexion torque	[36]
Shorter, K	Bidirectional pneumatic rotary actuator	1.9 Kg at the ankle, total weight 3.1 Kg	Provides 9 N·m plantarflexion torque	[38]
Noel, M	Electro-hydraulic system	Total weight 1.7 Kg	Provide 20 N·m auxiliary torque	[43]
Kim, S	Pneumatic components	0.5 Kg at the ankle, total weight 2.6 Kg	Provide 9.8 N·m plantarflexion torque	[44]
Choi, H	Artificial Pneumatic Muscle	1.44 Kg at the ankle, total weight 2.14 Kg	——	[46]

## Data Availability

Not applicable.

## Abbreviations

The following abbreviations are used in this manuscript:

AO	Ankle osteoarthritis
AFO	Ankle–foot orthosis
FES	Functional electrical stimulation
EC	Energy consumption
PAFO	Passive ankle–foot orthosis
SAFO	Semi-active ankle–foot orthosis
AAFO	Active ankle–foot orthosis
PNAAFO	Passive non-articulating ankle–foot orthosis
PAAFO	Passive articulating ankle–foot orthosis
ROM	Range of motion
COP	Center of pressure
FTS	Foot tilting strategy
DOF	Degree of freedom
